# Size‐dependence of food intake and mortality interact with temperature and seasonality to drive diversity in fish life histories

**DOI:** 10.1111/eva.13646

**Published:** 2024-02-07

**Authors:** Holly K. Kindsvater, Maria‐José Juan‐Jordá, Nicholas K. Dulvy, Cat Horswill, Jason Matthiopoulos, Marc Mangel

**Affiliations:** ^1^ Department of Fish and Wildlife Conservation Virginia Polytechnic Institute and State University Blacksburg Virginia USA; ^2^ Earth to Ocean Research Group, Department of Biological Sciences Simon Fraser University Burnaby British Columbia Canada; ^3^ AZTI, Marine Research, Basque Research and Technology Alliance (BRTA) Gipuzkoa Spain; ^4^ Instituto Español de Oceanografía (IEO‐CSIC), Centro Oceanográfico de Madrid Madrid Spain; ^5^ ZSL Institute of Zoology London UK; ^6^ Centre for Biodiversity and Environmental Research, Department of Genetics, Evolution and Environment University College London London UK; ^7^ Institute of Biodiversity, One Health and Veterinary Medicine University of Glasgow Glasgow UK; ^8^ Theoretical Ecology Group, Department of Biology University of Bergen Bergen Norway; ^9^ Institute of Marine Sciences and Department of Applied Mathematics and Statistics University of California Santa Cruz California USA

**Keywords:** body size, climate change, ecosystem size spectra, metabolic theory, state‐dependent models

## Abstract

Understanding how growth and reproduction will adapt to changing environmental conditions is a fundamental question in evolutionary ecology, but predicting the responses of specific taxa is challenging. Analyses of the physiological effects of climate change upon life history evolution rarely consider alternative hypothesized mechanisms, such as size‐dependent foraging and the risk of predation, simultaneously shaping optimal growth patterns. To test for interactions between these mechanisms, we embedded a state‐dependent energetic model in an ecosystem size‐spectrum to ask whether prey availability (foraging) and risk of predation experienced by individual fish can explain observed diversity in life histories of fishes. We found that asymptotic growth emerged from size‐based foraging and reproductive and mortality patterns in the context of ecosystem food web interactions. While more productive ecosystems led to larger body sizes, the effects of temperature on metabolic costs had only small effects on size. To validate our model, we ran it for abiotic scenarios corresponding to the ecological lifestyles of three tuna species, considering environments that included seasonal variation in temperature. We successfully predicted realistic patterns of growth, reproduction, and mortality of all three tuna species. We found that individuals grew larger when environmental conditions varied seasonally, and spawning was restricted to part of the year (corresponding to their migration from temperate to tropical waters). Growing larger was advantageous because foraging and spawning opportunities were seasonally constrained. This mechanism could explain the evolution of gigantism in temperate tunas. Our approach addresses variation in food availability and individual risk as well as metabolic processes and offers a promising approach to understand fish life‐history responses to changing ocean conditions.

## 1 INTRODUCTION

The body sizes and population biomasses of aquatic species are changing rapidly in response to human‐induced environmental change (Oke et al., 2020), motivating the need for mechanistic models to predict future patterns of growth and reproduction (Blanchard et al., 2012; Cheung et al., 2008, 2010; Fernandes et al., 2020). Beyond thermal physiology, the consequences of changes in ecosystem productivity for life history traits (growth, reproduction, and survival) will derive from changes in prey abundance, predation risk, and seasonality (Audzijonyte & Richards, 2018; Daufresne et al., 2009; Neubauer & Andersen, 2019). Classic and recent advances predicting optimal growth and reproduction of harvested populations (Beverton & Holt, 1959; Holt, 1958; White et al., 2022) have been combined with size‐structured community interactions to predict the evolution of life history traits in specific taxa (Shuter et al., 2016). This approach provides a promising foundation for predicting ecological and evolutionary responses to environmental and climate change. While specific taxa within an ecosystem will adapt according to contextual factors such as phylogenetic lineage, adaptive capacity, and environmental variability, mechanistic models incorporating the roles of size‐dependent foraging and predation risk, seasonality, and other metabolic processes could explain adaptive variation among closely related taxa and refine our mechanistic understanding of species' responses to changing environmental and climate conditions (Free et al., 2019). For example, by examining the life‐histories of closely related tuna species (family Scombridae) that have invaded different environmental niches, we can gain an insight into how physiological adaptations to thermal conditions, productivity, and seasonality in these factors affect allocation to growth and reproduction. This can help move us beyond broad macro‐ecological patterns toward a more mechanistic understanding of the drivers of existing diversity and improve predictions of species‐specific responses to future change (Álvarez‐Noriega et al., 2023).

In ectothermic species with indeterminate growth, including fish, food availability and survival both increase as individuals grow larger, and growth rates can be selected to be slower or faster depending on the size‐dependence of per‐capita resource availability and predation risk (Conover & Munch, 2002; Hulthén et al., 2021; Perrin et al., 1993; Walsh & Reznick, 2009). At the same time, development at higher temperatures can lead to maturation at smaller body sizes, within and between ectothermic species (Kingsolver & Huey, 2008). Allocation to growth and reproduction determine individual body sizes, fitness, and population demographic rates (Beverton & Holt, 1959; Gadgil & Bossert, 1970; Kozłowski, 1992, 1996). Yet existing theory struggles to predict how size‐dependent changes in prey availability and decreases in predation risk, along with interacting effects of metabolic costs and seasonality in food availability and temperature, affect the evolution of growth (Varpe, 2017; Yanco et al., 2022). There is longstanding interest in understanding the mechanisms leading to biphasic or asymptotic growth patterns (Quince et al., 2008). The earliest growth models, including the von Bertalanffy growth model, were based on hypothesized differences in the allometric scaling of anabolism (resources taken in) and catabolism (resources spent) (Audzijonyte et al., 2022; von Bertalanffy, 1960). Such growth models are routinely fit to data on size‐at‐age of fishes, but their mechanistic justification is necessarily simplistic; such models do not explain spatial and temporal trends in body size within and among species of fish (Audzijonyte et al., 2020), suggesting that they do not sufficiently capture the relevant intrinsic physiological and extrinsic ecological drivers of fish life histories.

The role of metabolic requirements and the trade‐off between growth and reproduction in shaping growth trajectories has been given increasing attention in recent decades (Jørgensen et al., 2016; White et al., 2022; Wong et al., 2021). Physiological processes can vary with the environmental temperature experienced by organisms (Brown et al., 2004; Clarke & Johnston, 1999). The allometric scaling of metabolic costs in different temperature regimes (known as the Metabolic Theory of Ecology [MTE]; Gillooly et al., 2001) has been used to predict individual body size according to the benefits of growing large (to increase foraging success and avoid predation), balanced against the costs of increased metabolic overhead and diverting resources from reproduction to growth (Thunell et al., 2023; Wong et al., 2021). More detailed dynamic models of energetic allocation have linked variable environmental conditions to growth, reproduction, and longevity (Audzijonyte et al., 2022; Cichoń & Kozłowski, 2000; Jørgensen & Fiksen, 2006; Kozłowski & Teriokhin, 1999; Lika & Kooijman, 2003). Understandably, the results of these energy budget models depend on specific assumptions regarding mass‐based foraging success and risk of predation. Generalizing these prior results requires a unified framework incorporating metabolic demands, access to resources, and predation risk in environments of differing productivity and seasonality.

**FIGURE 1:**
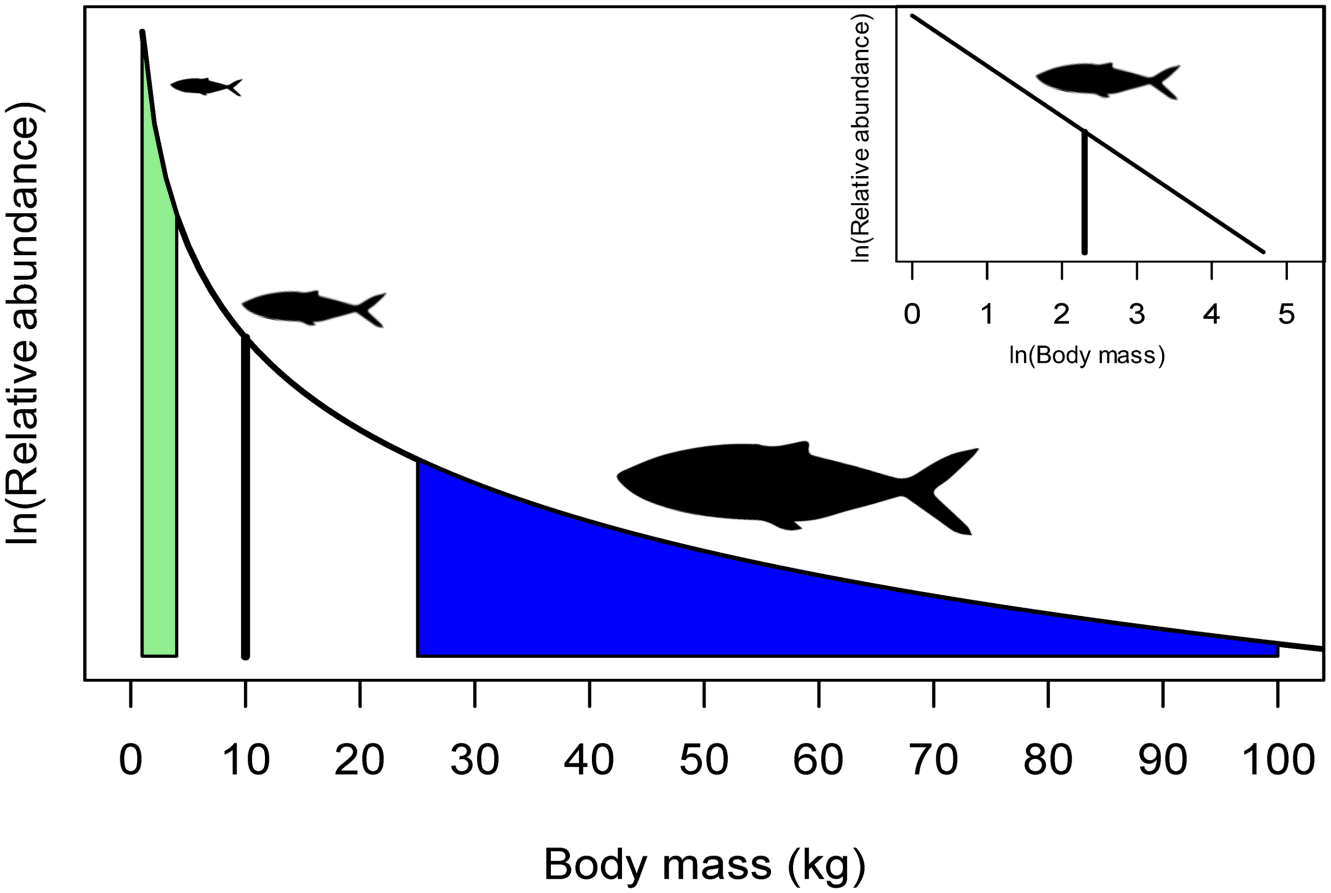
Conceptual version of the log‐linear size spectrum where the prey field of an individual of 10 kg (vertical black line) is in green and the predator field is in blue. The inset shows the same size spectrum in log–log space.

We draw on ecosystem size‐spectra theory to reduce the number of ad hoc assumptions required about the scaling of ecological processes that arise from community interactions. We hypothesize this approach could yield more realistic predictions of diversity in fish life histories, providing significant insights into the mechanisms that determine adaptive responses to changing environmental conditions. In aquatic ecosystems, size spectra are an emergent property of community interactions (Andersen, 2019; Andersen et al., 2016; Christensen & Andersen, 2011; Law et al., 2009; Sheldon et al., 1977; Sprules & Barth, 2016; Thygesen et al., 2005). In a community size spectrum, flow of energy between trophic levels via consumption and predation rates are characterized by individual mass, instead of species' identity (Andersen, 2019; Benoît & Rochet, 2004; Blanchard et al., 2009). The key property of size spectra is that an individual's relative position (mass) determines both its prey field (the area under the size spectrum to the left of an individual's mass) and its risk of predation (the area to the right) (Figure 1). That is, the size spectrum provides a simplified quantification of predator–prey interactions for a given individual in an ecosystem. Individuals are born small and grow through the size spectrum over their lifetime, consuming prey that are a fraction of their own size. At the same time, the number of predators that are capable of consuming an individual decreases as the individual increases relative to the number of predators capable of consuming it because size‐spectrum theory assumes predators cannot consume prey exceeding their maximum gape. By contrast, the lower limit of prey size preference is assumed to depend on the profitability of the prey. These interactions apply to interactions between species as well as dynamics within size‐structured populations of the same species (i.e., species of fish often cannibalize smaller conspecifics). Therefore, predation and consumption rates determined by different areas under a size‐spectrum curve can be used to simultaneously characterize the mass‐specific caloric resource availability and risk of predation experienced by an individual as it grows (Andersen, 2019; Benoît & Rochet, 2004; Giacomini et al., 2013; Shuter et al., 2016).

Our first objective was to develop an energetic model of optimal allocation to growth and reproduction that accounts for size‐dependent resources and risk of predation (both derived from size‐spectra) and size‐ and temperature‐dependent metabolic requirements. We hypothesized that such a model can predict the evolution of diverse fish life histories in different environments, based on these allometric relationships. Fitness was defined as the expected lifetime reproductive output. Optimal life history strategies (allocation to maintenance, growth, and reproduction) were then determined by maximizing fitness using stochastic dynamic programming (Clark & Mangel, 2000; Houston et al., 1988; Mangel, 2015). We used this approach to predict the life history evolution of fishes under different scenarios of ecosystem productivity and temperature via their effects on the size‐spectrum and metabolic costs.

Our next objective was to determine if our modeling approach could predict the evolution of growth, body sizes, and reproductive patterns as observed in tuna species under several environmental scenarios with varying temperatures, ecosystem productivities, and seasonally varying conditions. Tunas (family Scombridae) exhibit a wide range of maximum body sizes (~40–400 cm), longevities (~4–41 years), and reproductive patterns (Horswill et al., 2019; Juan‐Jordá et al., 2013). Tunas are epipelagic species found in temperate and tropical waters around the world's oceans with varying vertical, latitudinal, and seasonal distributions and movements. Confronting the predictions of our general model with data on the life history diversity observed in tuna species in different environmental conditions provided an insight into the mechanisms underlying fish life histories. Where possible, our models were parameterized with values for physiological processes measured for tunas, but we also considered the sensitivity of our results to these parameters to ensure their generality across the diverse life histories observed in fishes.

## 2 METHODS

We used size‐spectrum theory to infer mass‐specific rates of prey encounter and mass‐specific rates of predation. The relationship between the numbers of organisms *N* in the ecosystem and individual mass *w* is a power function with a scaling parameter κ and an exponent λ. This exponent has been empirically estimated to be close to −1, such that abundance is inversely proportional to mass (Hatton et al., 2021; Sheldon et al., 1972; Sprules & Barth, 2016; Trebilco et al., 2013)

(1) $$N\left(w\right)=\kappa w^{\lambda }$$

The Sheldon size spectrum, which follows from Equation 1, is the distribution of total ecosystem biomass *B*(*w*) across body size classes and is represented by $B\left(w\right)=N\left(w\right)w.$ Based on evidence from multiple ecosystems (Sprules & Barth, 2016), the spectrum – as originally conceived (Sheldon et al., 1972) and recently confirmed (Hatton et al., 2021) – is nearly flat or may very slowly decrease as body size increases. Since $N\left(1\right)=\kappa$, we view κ as a metric of ecosystem productivity, which in our model we assume is in units of kilograms per month. In a log–log plot of Equation 1, the intercept is log(κ).

This phenomenon of linear aquatic size spectra emerges from three size‐dependent processes: (1) the encounter rate of predators and prey; (2) the preference of predators for prey of a given size; and (3) the limit to prey consumption imposed by the size of the predator's stomach (the predator–prey mass ratio; Andersen, 2019; Benoît & Rochet, 2004; Blanchard et al., 2017).

Here, we follow the modeling work of Andersen (2019), which derived a method to calculate the prey available to individuals of mass *w* by relating the productivity of a spectrum to empirical estimates of prey encounter rates, predator–prey mass ratios, and prey preferences. Andersen (2019) assumed that the prey biomass available to an individual is a concave function of mass *w* and found it scaled to be approximately three times the size spectrum productivity κ. The empirical estimates of physiological processes used to simplify this derivation can vary among species within a size class (Andersen, 2019). Therefore, we assume that prey availability and individual consumption are proportional such that the per‐period food available for an individual of mass *w* is represented by

(2) $$B_{\text{prey}}\left(w\right)=3\kappa w^{0.05}$$

**FIGURE 2:**
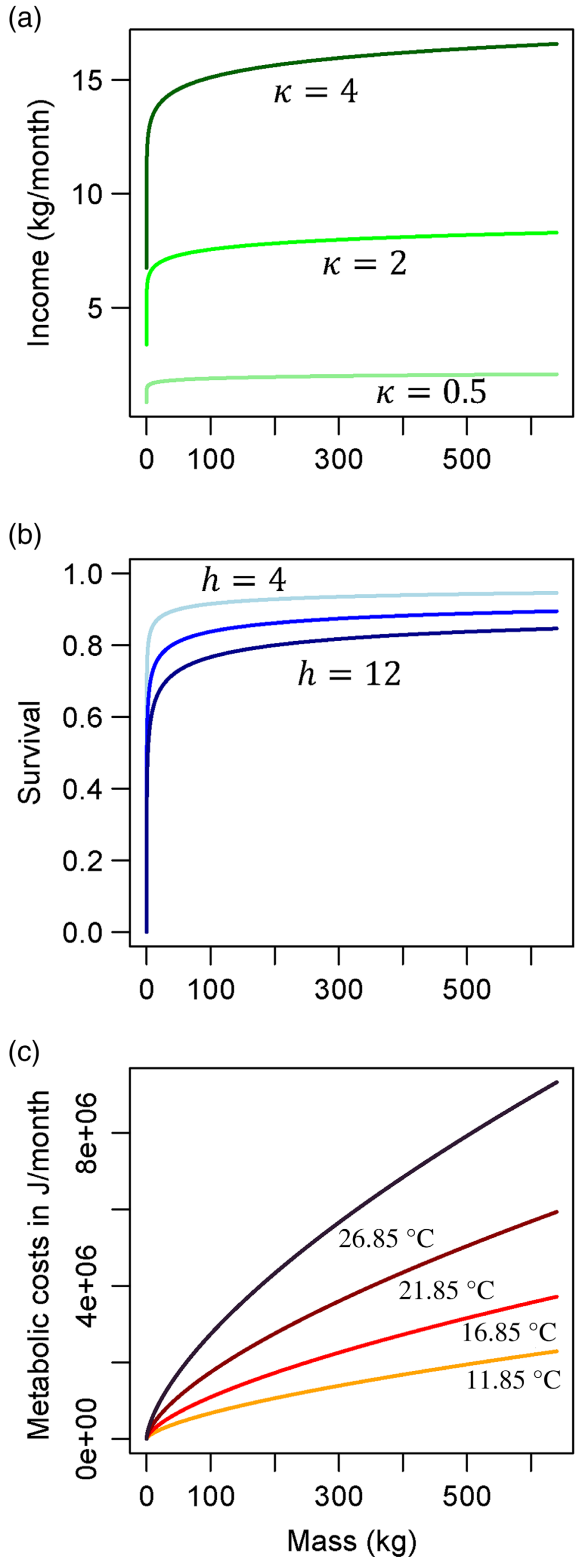
Examples of allometric relationships between individual mass and (a) income or energy gained (in kg per month) from prey biomass for differing ecosystem productivities κ (Equation 2); (b) the scaling of survival (the inverse of the size‐based risk of mortality via predation) and its interaction with predator *h* (Equation 4), such that higher values of *h* represent more efficient predators; and (c) metabolic costs (Equation 5), which also scale with temperature τ (on the graph, it is presented in degrees Celsius, °C; values in Kelvin are 285, 290, 295, and 300 K). Note that these curves represent a subset of values considered in our results.

This equation approximates the integral over the range of prey biomass (in kg) available to an individual of mass *w* each month (Figure 2a) and includes the threefold scaling factor from empirical analyses (Andersen, 2019). The prey field is therefore the total biomass available to the predator and the range of sizes of prey that it takes. This allometry is very different from the functional forms assumed in the von Bertalanffy growth model or Dynamic Energy Budget Theory. To address variability across ecosystems in productivity, we chose to vary κ in subsequent analyses, representing potential differences among taxa or other ecosystem processes.

Next, we calculate survival per time from the mass‐specific risk of predation that emerges from size‐spectrum theory. We assume that the processes determining consumption rates of predators in the size spectrum can be used as a proxy for individuals' instantaneous rate of mortality from predation, following the derivation in Andersen (2019). This derivation is based on first principles. For gape‐limited taxa like fishes, a predator's prey field depends on its encounter rate with prey in its preferred size range. This encounter rate (and the clearance rate) can be measured in units of volume per time, as aquatic species forage in a three‐dimensional habitat (Kiørboe & Hirst, 2014). The risk of mortality from a single predator is therefore the volume of prey cleared by a predator, relative to the volume encountered, and weighted by the size of its preferred prey. This must be multiplied by the density of predators and integrated over all sizes (box 2, Andersen, 2019). For simplicity, we ignored the potential effects of temperature on encounter or clearance rates that could arise from an increase in activity associated with warmer environmental conditions. In Equation 3 (defined below), we used an empirically estimated constant of 0.07 to characterize the scaling of prey vulnerability with its mass (Andersen, 2019), which is based on estimates of preference windows of predators and the volume of water each clears per month that comes from empirical distributions of prey sizes in predator guts (Ursin, 1973). We also define a coefficient *h*, which modifies the probability of consumption – how likely a predator is to capture the focal individual (based on its capture efficiency) – and used the reported allometric exponent of −0.25 to represent how predation mortality scales with body size (Figure 2b; eq. 2.11 in Andersen, 2019). Andersen (2019) integrated over the Sheldon spectrum to produce a relationship predicting the scaling of mortality risk for prey $\mu_{p}\left(w\right)$ per month:

(3) $$\mu_{p}\left(w\right)=0.07hw^{-0.25}$$

We convert this instantaneous rate to the probability of escaping from predators during each time interval (Hilborn & Mangel, 1997; Mangel, 2006), so we can represent the probability an individual survives $\gamma_{\text{pred}}\left(w\right)$ as

(4) $$\gamma_{\text{pred}}\left(w\right)=e^{-\mu_{p}\left(w\right)}$$

The resulting mass‐specific survival through each month is plotted for different values of *h* in Figure 2b.

### 2.1 Defining metabolic costs that depend on temperature and body mass

Our energetic model includes metabolic costs which depend on temperature and body mass. We assumed that metabolic costs increase with body mass and environmental temperature (Clarke, 2006). We modelled individual costs $C\left(w,\tau \right)$ (in joules) as a function of temperature 𝜏 in Kelvin, following the general form introduced in the MTE (Gillooly et al., 2001):

(5) Cwτ=cwθe−EkBτ

Evidence for the MTE suggests the activation energy *E* (the energy required for the reactions of respiration and other metabolic processes) varies little among taxa (Bernhardt et al., 2018; Brown et al., 2004, but see Lindmark et al., 2022); Boltzmann's constant $k_{B}$ also does not vary. The normalization coefficient *c* accounts for differences among taxonomic groups in the intercepts of the linear relationship that arise from second‐order effects such as stoichiometry or respiratory surface areas (Bigman et al., 2021). The slope of this relationship in log space, *θ*, is strikingly similar among taxa (Brown et al., 2004). We used a value for *θ* estimated from physiological studies on tunas (Table 1) but varied it in sensitivity analyses. Note that τ in Equation 5 is traditionally in Kelvin. However, we rescale all values in our results in units of Celsius. We use this general formula for scaling of metabolic costs at different temperatures τ to describe the monthly energy expenses of an individual of mass *w* (Figure 2c). Defining the relationships in Equations 2, 4, and 5 allowed us to specify mass‐dependent survival and energy dynamics and therefore examine the variables influencing growth and reproduction in a common currency of individual fitness. All parameters are defined and their values reported in Table 1.

**TABLE 1:** Description of parameters and variables.

Parameter or variable	Description	Value
*w*	Body mass in kg	–
*N*	Number of individuals in a size category (or trophic level) when considering a Sheldon size spectrum	–
*B*	Absolute biomass in a size category (or trophic level) when considering a Sheldon size spectrum	–
κ	The spectrum parameter, which defines the total biomass of organisms of the smallest body size *w* in a given ecosystem; Andersen (2019) gives an estimate of 10 gained by averaging over all Predator–Prey Mass Ratio estimates measured from gut contents. We vary it to represent ecosystem differences in overall ecosystem productivity	Varies
*B*_prey_	Biomass of prey expected by a focal individual	–
*μ*_*p*_	Instantaneous risk of mortality due to predation, which depends on body mass and position in the size spectrum	–
h	Predation risk, comprising predator satiation estimates (estimated from gut contents) and predator preference (or effectiveness) for consuming prey of a given mass (Andersen, 2019)	4, 8
𝜏	Temperature of the environment (in degrees Kelvin except where noted)	285–300
*C*	Metabolic requirements (costs) that scale with mass and temperature	–
*c*	Normalization constant scaling metabolic costs (in J), based on metabolic rate data from tunas (Kitchell et al., 1978)	5 × 10^16^
$k_{B}$	Boltzmann constant, relating particle energy to temperature in units of m^2^ kg s^−2^ K^−1^	1.3 × 10^−23^
*E*	The average activation energy for the rate‐limiting enzymes in metabolism in units of joules; from the metabolic theory of ecology (Brown et al., 2004).	1.04 × 10^−19^
𝜃	Metabolic scaling exponent; values vary among clades; here we use a value reported for tunas (Clarke & Johnston, 1999)	0.66
𝜌	The energy density of tuna body mass in our model in J/kg (estimated empirically and reported in Chapman et al., 2011)	4.2 × 10^6^
*t*	Time in monthly time steps in the dynamic model	–
*T*_max_	Maximum lifespan in months	216
*l*, *L*(*t*)	Body length (in cm) This is a dynamic state variable but can only increase with time. The maximum value possible is 400 cm. For a specific value, we use the capital letter notation	–
*s*, *S*(*t*)	Lipid stores (in J) – this is a dynamic state variable representing energy stores that can be used for metabolism, growth, and reproduction	–
*a*	Scale coefficient relating length to structural mass, similar to values estimated empirically for bluefin tuna and reported in Pignalosa et al. (2020)	1.0 × 10^−5^
$w\left(t\right)$	Structural mass of the individual (in kg) at time *t* – this depends on *L*(*t*)	–
υ	The fraction of structural mass that determines the critical threshold of energy mass needed to avoid starvation	0.1
*φ*	The fraction of structural mass that determines the maximum possible reproductive output in a monthly time step	0.2
$\gamma_{\text{pred}}$	Survival from predation from 1 month to the next, emerging from risk of mortality	
$\gamma_{s}$	Survival, given that the individual has sufficient energy reserves to meet metabolic requirements and avoid starvation	
*g*	Proportion of lipid stores allocated to growth (this allocation decision is optimized by the dynamic programming equation); can take values between 0 and 1	
*r*	Proportion of lipid stores allocated to reproduction (this allocation decision is optimized by the dynamic programming equation); can take values between 0 and 1 and the sum of *g* and *r* cannot exceed 1	
$R\left(l,s,t\right)$	Current fitness, the product of lipid stores and the optimal allocation to reproduction, *r*	
$V\left(l,s,t\right)$	Expected accumulated lifetime reproduction from time t onward, given that $L\left(t\right)=l$ and $S\left(t\right)=s$	

We note that environmental temperatures can also affect the encounter rate of prey through its effects on activity level. Additionally, productivity increases with temperature, as it elevates the rates of consumption and respiration, improving growth rates (Audzijonyte et al., 2022). We have chosen to address these potential interactions between temperature and energy intake by comparing growth patterns that occur at the same temperatures with differing levels of ecosystem productivities, such that the potential relationship between κ and temperature is implicit.

### 2.2 Defining fitness and determining the optimal allocation strategy

**FIGURE 3:**
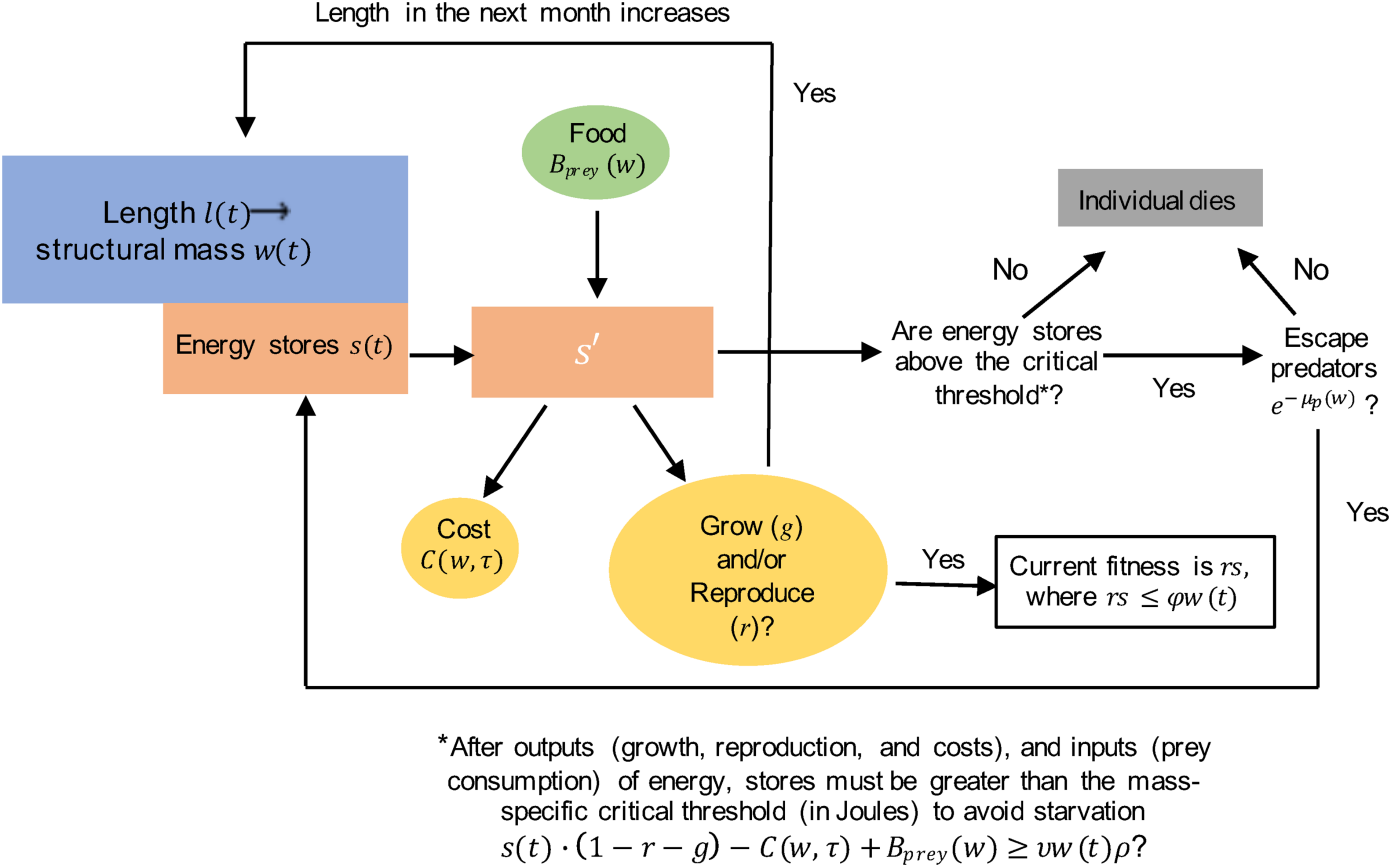
Conceptual overview of the optimization algorithm calculating the two dynamically varying state variables, body length $l\left(t\right)$ and energy stores $s\left(t\right),$ within each month of an individual's life *t*, as well as current and expected fitness. Arrows represent the flow of energy or decisions. Round shapes represent energy input (green) and outputs (yellow); rectangular shapes are model states and outputs (fitness and fate). Note that $l\left(t\right)$ and $w\left(t\right)$ are related through Equation 6. Both *B*_prey_ and $\mu_{p}$ are determined by the size spectrum.

To find the optimal allocation strategy in different scenarios of ecosystem productivity and temperature conditions, including seasonal variation in the scenarios, we developed a model of an individual's energy budget, tracking two physiological state variables, the body length *l* and energy (lipid) stores *s* of an individual, which vary dynamically over an individual's lifetime.

(Figure 3). Following the conventions for dynamic state‐variable models, we denoted the state variables *l* and *s* as lowercase in the dynamic programming equation, representing the fact they are iterated values; potential future states are denoted as *l*′ and *s*′. Later, when we refer to values of the state variables at a specific time, we use uppercase *L* and *S*.

We define fitness as expected lifetime reproductive output, averaged over the stochastic process of mortality from both predation and starvation, which we calculated numerically using stochastic dynamic programming (Clark & Mangel, 2000; Houston et al., 1988; Mangel, 2015). This method allowed us to consider how individual age and physiological state (energy stores and body length) affect the optimal trade‐off between growth and reproduction in the context of expected lifetime reproductive output. We assumed that an individual allocates a proportion of its budget to growth and reproduction on monthly time intervals. Our choice to model allocation to growth and reproduction as proportions of an individual's energy budget builds on prior dynamic state‐variable models of fish growth (Chapman et al., 2011; Jørgensen & Fiksen, 2006).

Given specific values of $L\left(t\right)$ and $S\left(t\right)$, representing individual length and energy stores at the start of month *t*, structural mass $W\left(t\right)$ is related to length with a standard cubic function (Froese, 2006):

(6) $$W\left(t\right)=\mathrm{aL}{\left(t\right)}^{3}$$

We assumed that only structural mass is relevant for size‐dependent gains (*B*_prey_) and costs (*C*) (Figure 3). We convert from mass to units of energy (joules) and back using a conversion factor estimated for tunas ($\rho =4.2\times 10^{6}$ J/kg; Chapman et al., 2011) but that could take other values for other species. Each month, an individual acquires energy from food, determined by their structural mass *w* via Equation 2. We then allowed the individual to allocate proportions of its energy budget (which includes income and any stored energy) to growth (*g*) and reproduction (*r*).

Given an individual with specific values for length $L\left(t\right)$ and energy stores $S\left(t\right)$, we calculated the increment of growth expected for each possible proportional allocation *g* by converting the fraction of lipid stores from joules to the equivalent mass $\left(g\frac{S\left(t\right)}{\rho }\right)$ and then adding it to existing mass, such that $W\left(t+1\right)=W\left(t\right)+g\frac{S\left(t\right)}{\rho }$. We then calculated the new length by rearranging the mass–length relationship $W\left(t\right)=\mathrm{aL}{\left(t\right)}^{3}$:

(7) $$L\left(t+1\right)={\left(L{\left(t\right)}^{3}+g\frac{S\left(t\right)}{\mathrm{a\rho}}\right)}^{1/3}$$

We repeated this for every possible combination of values of the state variables, *l* and *s*. Values for proportional allocation of lipid stores to reproduction *r*, along with *g*, combine to determine the dynamics of energy stores and length from 1 month to the next:

(8) $$S\left(t+1\right)=B_{\text{prey}}\left(W\left(t\right)\right)-C\left(W\left(t\right),\tau \right)-\left(r+g\right)\cdot S\left(t\right)$$

We assumed that both stored energy and reproduction were limited by an individual's structural mass (which in turn depends on its length). These constraints represent limits on both the amount of lipid that can be stored and the mass of gametes that can be produced, given the capacity of the body cavity. If the proportions of energy allocated to reproduction and growth were less than 100% (meaning r+g<1), the remaining energy was assumed to be stored for future use, given that total reserves did not exceed 60% of structural mass. This value was arbitrary, but exploratory analyses suggest it did not have a strong effect on the results presented here because in practice individuals do not store their energy long enough to come close to exceeding this limit. Reproductive output (in units of kg) was similarly constrained so that it cannot exceed a fixed proportion of φ of structural mass $w\left(t\right),$ such that the following condition must be met:

(9) $$\frac{\mathrm{rS}\left(t\right)}{\rho }\leq \mathrm{\varphi w}\left(l,t\right)$$

This size‐based limit on total reproduction is used in the calculation of current fitness.

### 2.3 Expected future fitness

At any age, expected fitness was the sum of current reproduction and accumulated future reproduction, which was calculated assuming the individual behaved optimally for all future ages. This required calculation of the future states (length and lipid stores) given each combination of allocation to growth and reproduction. We denoted potential values of future states as $l^{\prime }$ and $s^{\prime }$. These are

$$l^{\prime }\left(l,s,g\right)=l+{\left(l^{3}+g\frac{s}{\mathrm{a\rho}}\right)}^{1/3}$$

(10) $$s^{\prime }\left(l,s,r,g\right)=B_{\text{prey}}\left(w\left(l\right)\right)-C\left(w\left(l\right),\tau \right)-\left(r+g\right)s$$

where $w\left(l\right)$ is the structural mass of an individual of length *l*. At the end of the month, if an individual's lipid stores fell below the critical threshold for its mass (its expenditures have exceeded its energy budget), it starved (Figure S2). We let $\gamma_{s}\left(s,l\right)$ denote the probability of avoiding starvation in the current time and modelled it as

(11) $$\gamma_{s}\left(s,l\right)=\frac{1}{1+e^{-q\left(s-\mathrm{\upsilon w}\left(l\right)\rho \right)}}$$

involving two new parameters υ and *q*. The parameter υ determined the level of stores at which starvation begins; *q* is a shape parameter. When $s=\mathrm{\upsilon w}\left(l\right)\rho$, the right‐hand side of Equation 11 is always ½. When *q* is large, then the right‐hand side of Equation 11 is approximately 1 when $s>\mathrm{\upsilon w}\left(l\right)\rho$ and 0 otherwise. Thus, this function specified that if combined allocation to growth and reproduction caused an individual's lipid stores to decrease below the critical value for its mass, its probability of survival decreased smoothly toward 0 (see Figure S1 for more details).

We varied growth *g* and reproduction *r* and determined the combination that maximized fitness. Equations (7), (8), (9), (10), (11) define changes in individual state and in fitness for all allocation strategies (all values *r* and *g*). With these functions in place, we can find the allocation strategy that maximizes current and future fitness at every age until the age of senescence or maximum lifespan, *T*, is reached (for all scenarios, we assumed the maximum lifespan of *T =* 216 months or 18 years). We define $V\left(l,s,t\right)$ as the maximum expected accumulated reproduction between time *t* and *T*, given size $L\left(t\right)=l$ and lipid stores $S\left(t\right)=s$. Since there can be no accumulated reproduction after *T*, we assumed $V\left(l,s,T\right)=0$. Expected future fitness at every age *t* < *T* was found by solving the stochastic dynamic programming equation, which for all values of allocations *r* and *g* and age in months *t* decomposes expected reproduction from time *t* onwards into reproduction at time *t* and expected reproduction from time t+1 onward given the new values of the states:

(12) $$V\left(l,s,t\right)=\max_{g,r}\left[R\left(r,l,s,t\right)+\gamma_{\text{pred}}\left(w\right)\cdot \gamma_{s}\left(s,l\right)\cdot V\left(l^{\prime }(l,s,g),s^{\prime }\left(l,s,r,g\right),t+1\right)\right]$$

The first term on the right‐hand side of Equation 12 represents reproduction in month *t*. The second term represents expected future reproduction, discounted by the probability of surviving predation $\gamma_{\text{pred}}$ $\left(w\right)$ and starvation $\gamma_{s}\left(s,l\right)$. When these are combined, we can obtain expected lifetime reproduction from time *t* onward, given that size $L\left(t\right)=l$ and lipid stores $S\left(t\right)=s$.

The dynamic programming algorithm (Houston & McNamara, 1999; Mangel & Clark, 1988) iterates over all viable combinations of *l* and *s*, at each time *t*, and stores the fitness of each allocation strategy. The optimal strategy (marked with an asterisk) at time *t* is the combination g* and r* associated with the greatest current and future fitness. Further details of the optimization algorithm are given in the Appendix A. In Figure S3, we illustrate the array for both allocation strategies (g*(*l*, *s*, *t*) and r*(*l*, *s*, *t*)) at two ages, for all possible combinations of length and lipid stores.

### 2.4 Calculating the fates of a cohort of individuals allocating optimally

Assuming that an individual followed optimal allocations determined by Equation 12, we specified the length at birth and used forward iteration (Clark & Mangel, 2000) to determine the accumulated mortality and reproductive output as a function of time. Some combinations of states (length, lipid stores, and age) will not arise naturally, and others are inviable (the dark blue regions of Figure S3). We recorded body lengths and reproductive outputs in subsequent months and calculated the probability of survival to age, given both the risk of starvation and the risk of predation. We defined expected lifespan as the age past which expected survival was less than 3%. For simplicity, we considered reproductive output in units of energy (joules) rather than considering allocation to offspring size and number (Kindsvater et al., 2010); this can include migration costs. We did not build in any assumptions about age or size at maturation, but rather let maturation patterns, along with natural trade‐offs between growth and reproduction (Figure S3), emerge from patterns of allocation.

### 2.5 Scenarios for environmental variation

We used our general energetic model to ask whether we could predict a range of fish life histories (patterns of growth, reproduction, and lifespan) that are evolutionarily advantageous across different scenarios of ecosystem productivity and environmental temperatures. To do this, we developed different productivity and temperature scenarios corresponding to different conditions that impose different metabolic costs to individuals according to Equation 5. We solved for the optimal life histories under different environmental temperatures (τ in Equation 5 in units of Kelvin), converted to Celsius and ranging from 11.85 to 26.85°C (285–300 K) in 5° intervals. Temperature affected energy budgets of individuals but did not directly affect consumption rates, which increase in warmer conditions (Clarke, 2006). To tease apart the effects of thermal costs from temperature‐effects on resources, we considered these temperature ranges in different scenarios for ecosystem productivity, for which κ ranged from 0.25 to 5, in factorial combinations.

### 2.6 Case study predicting life history diversity of tunas (Scombridae)

We asked if our general energetic model predicted the patterns of life history variation observed in tunas, a group of species adapted to different environments. There are 15 species of tunas within the Family Scombridae, from five genera: *Allothunnus*, *Auxis*, *Euthynnus*, *Katsuwonus*, and *Thunnus* (Collette et al., 2001). These species inhabit a wide range of environmental conditions in marine ecosystems. Paleo‐oceanographic evidence suggests that ancestral tunas evolved in a tropical environment approximately 60 million years ago (Monsch, 2000), and over time they have diversified and evolved a suite of morphological and physiological adaptations that have allowed them to expand their distributions into more temperate environments or deeper colder waters where they can encounter higher prey densities to support their high somatic and gonadal growth rates (Dickson & Graham, 2004). Currently, tunas can be found in coastal and oceanic pelagic waters and have wide geographic distributions, ranging from the tropics to higher temperate latitudes with some degree of habitat partitioning by depth. Tropical tunas can spawn throughout the year, while the subtropical and temperate tunas undergo seasonal migrations returning from cool high‐latitude feeding grounds back to warm waters for spawning (Horswill et al., 2019; Juan‐Jordá et al., 2013). Reflecting their tropical ancestor, all tunas (except for the slender tuna *Allothunnus fallai*) spawn in warm waters, requiring a sea surface temperature of at least 24°C (Schaefer, 2001). This is a key aspect of their reproductive biology that we include in our model scenarios.

**FIGURE 4:**
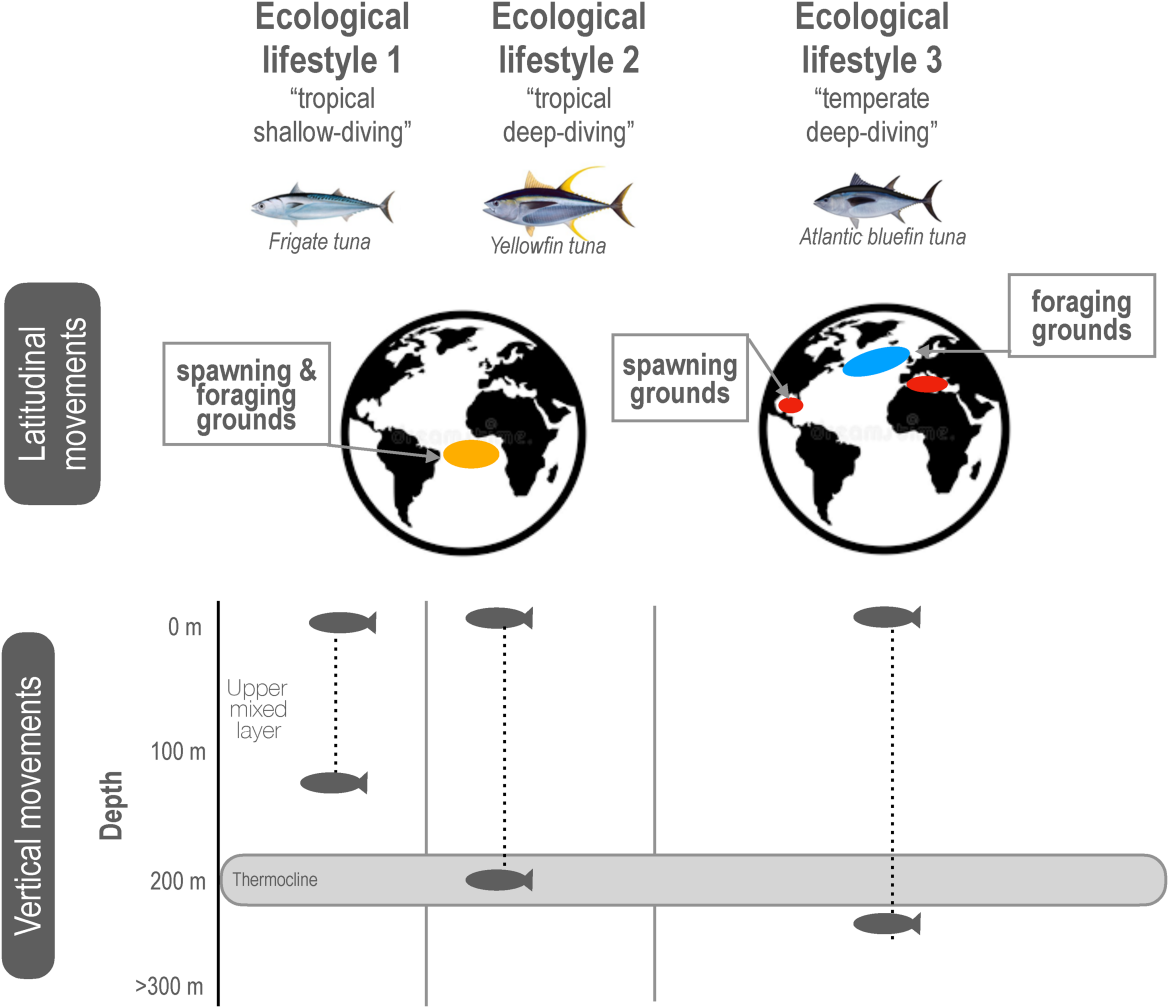
Three representative ecological lifestyles of tunas and their distribution patterns based on their latitudinal and vertical movements. Characteristic spawning and foraging grounds are shown for each lifestyle; The three ecological lifestyles illustrate a tropical shallow‐diving frigate tuna *Auxis thazard*, a tropical deep‐diving yellowfin tuna *Thunnus albacares*, and a temperate deep‐diving Atlantic bluefin tuna *Thunnus thynnus*. Fish silhouettes represent the depth distribution where species spend most of their time. The thermocline is defined as the depth range within which the water temperature changes rapidly and separates the water column into the upper well‐mixed surface layer (water above 20°C) and the deeper waters (waters below 15°C). Figure modified from Bernal et al. (2009) and (2017).

To connect our general energetic model more explicitly with the observed patterns of life history variation in tunas, we followed a proposed categorization of tunas into three ecological lifestyles (Bernal et al., 2017). The three general lifestyles are based on species‐specific vertical, latitudinal, and temporal (seasonal) distributions and movement patterns of tunas (Figure 4; Bernal, 2011; Bernal et al., 2009, 2017). The first ecological lifestyle represents a tuna species that largely remains within the warmer and well‐oxygenated surface layer above the thermocline (generally above 20°C) during both day and night. These tuna species have limited vertical movements as they do not descend below the thermocline (Figure 4). Coastal species, such as the tropical frigate tuna (*Auxis thazard*), may typify this group. The second ecological lifestyle represents tuna species that spend the majority of the time above the thermocline (generally above 20°C) but also visit depths below the thermocline for foraging (Figure 4). The oceanic species of yellowfin tuna (*Thunnus albacares*) with year‐round tropical distributions typifies this group. Its vertical movement exposes this species to a wider range of temperatures and to less‐oxygenated waters at depth but only for short periods of time because this species is not hypoxia‐tolerant (Schaefer et al., 2009). The third ecological lifestyle characterizes tuna species that are exposed to a wider range of environmental conditions and spend significant periods of time in colder waters (Figure 4). The oceanic and temperate Atlantic bluefin tuna (*Thunnus thynnus*) are one species that typifies this group, spending most of the year at higher latitudes in colder and more productive waters between the upper mixed later and the cooler deep waters below the thermocline and migrating to warmer waters for spawning (Bernal et al., 2017).

To examine whether our general model could predict these three broad ecological lifestyles of tunas, we modelled environmental scenarios that correspond to the habitat of each representative species in terms of temperature, ecosystem productivity, and seasonality (Table 2). We chose parameters based on the results of our general model but also added seasonal fluctuations in environmental temperatures (which increased metabolic costs) and productivity to represent the bluefin tuna migration from temperate, productive waters to their tropical spawning grounds. We then asked whether the life‐history traits emerging in these scenarios are consistent with the range of reported sizes of three representative species corresponding to each lifestyle (Juan‐Jordá et al., 2016). This analysis assumes that our model assumptions regarding the relationship between temperature and metabolic costs, which is derived from macro‐ecological patterns (Gillooly et al., 2001), holds for closely related species of tunas. Further, note that we do not have direct information on values of κ in different oceanic environments. Variation in κ in this analysis could represent positive effects of temperature on overall resource availability as well as consumption.

**TABLE 2:** Parameter values for environmental scenarios corresponding to the three ecological lifestyles of tunas described in Figure 3, with corresponding predicted body size and maximum observed fork length (cm) for three representative species (data from Juan‐Jordá et al., 2016).

Lifestyle	Environment	Baseline or winter temp	Yearly mean κ	*h*	Predicted body size	Representative species	Reported range of body sizes
Tropical shallow	Constant	26.85°C	0.1	8	62 cm	Frigate tuna	40–60 cm
Tropical deep diving	Constant	21.85°C	1	8	214 cm	Yellowfin tuna	125–231 cm
Temperate deep diving	Seasonal	11.85°C	2.5	12	361 cm	Bluefin tuna	203–427 cm

### 2.7 Sensitivity analyses

We ran a series of tests to examine how our choices of parameters in the size spectrum influence model predictions. Specifically, we varied values of *h* (representing efficiency in prey capture) in the function describing the risk of mortality (Equation 3) and φ (the fraction of body mass that can be devoted to reproduction) in the reproductive constraint (Equation 9). We also conducted sensitivity analyses in which we varied the scale and shape coefficients in the metabolic cost function (*c* and θ, respectively, in Equation 5). Motivated by our tuna case study, we also varied seasonality in resources, thermal (metabolic) costs, and spawning. We considered seasonal environments in which only resources and only thermal costs varied to understand how these factors alone contributed to observed variation in body size and reproductive output. We additionally considered growth and reproductive patterns with an extended warm season (where individuals could spawn in warm temperatures for 6 months of the year, instead of 3). Finally, we determined in preliminary analyses that the maximum lifespan *T* did not strongly affect model results because most individuals reach a maximum body size well before this threshold.

## 3 RESULTS

**FIGURE 5:**
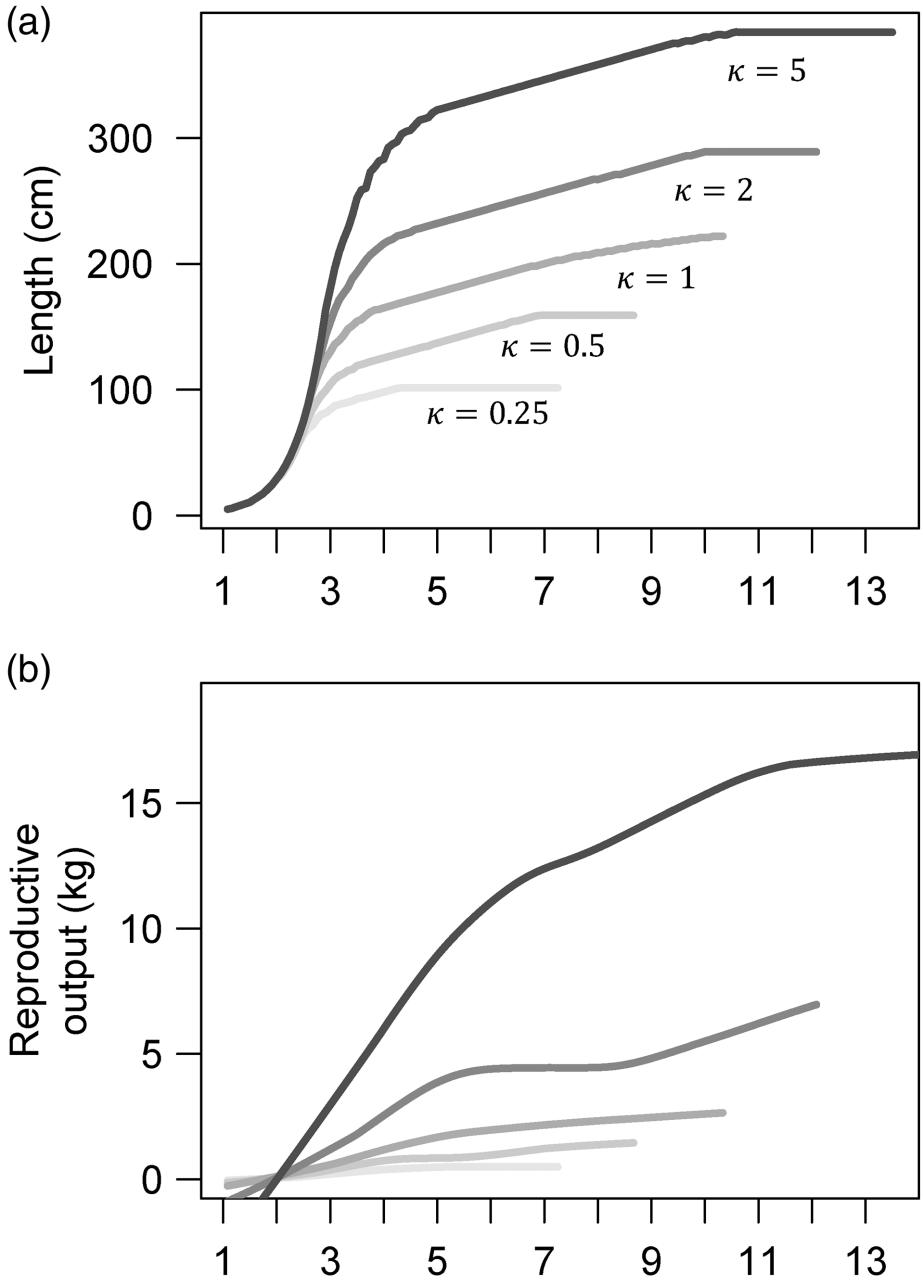
(a) Growth (length at age) and (b) expected reproductive output of individuals in different productivity κ scenarios. Each curve ends at the age (maximum lifespan) at which an individual's cumulative chance of mortality due to predation or starvation is greater than 97%. Temperature in every scenario was constant at 16.85°C, and *h* was constant at 8. The reproductive output was smoothed using the loess tool in R; raw data points can be found in Figure S4.

An asymptotic growth pattern naturally emerged from the model, after a period of exponential growth early in life (Figure 5). Our model predicted age‐specific relationships between body size (length, from which we calculate mass using Equation 6) and reproduction that correspond to a range of fish life histories (Figure 5). In general, we found that individuals allocated energy to growth early in life and shifted this allocation to reproduction in later life. Ecosystem productivity (κ) alone generated a range of maximum body sizes, from less than 100 cm at low levels of productivity to well over 350 cm (Figure 5a). Across scenarios of ecosystem productivity, the predicted trajectories for individual growth were identical in early life (before age 2); subsequent growth slowed earlier in lower‐productivity environments (Figure 5a). For all life histories, allocation to reproduction began at very low levels sometime during the individual's second year (Figure 5b) and increased steadily as the individual aged. Both the rate at which reproduction increased with size and the maximum reproductive output were consistently greater with increased ecosystem productivity (Figure 5b). We also found the pattern of age‐specific mortality was very similar in early life for all productivities κ, but that lifespan increased predictably as asymptotic body size increased (Figure S5).

**FIGURE 6:**
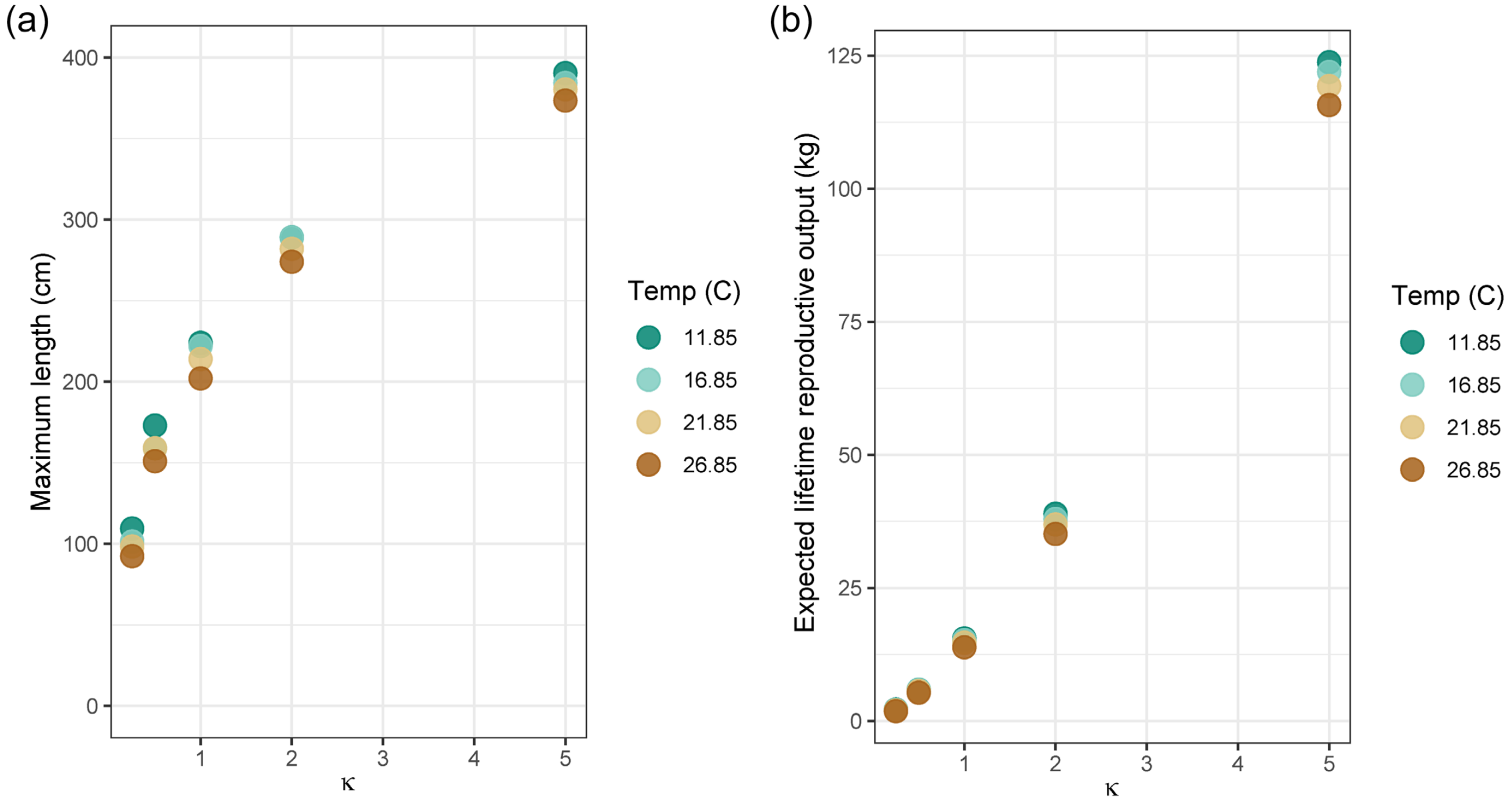
(a) Maximum body size and (b) expected lifetime reproductive output (in kg) of individuals plotted against a range of productivity scenarios. Color of points corresponds to differences in average annual temperature in °C, representing increased physiological costs.

When we compared the interacting effects of ecosystem productivity (κ) and metabolic costs of elevated temperature (τ) on patterns of growth and reproductive allocation, we found the effects of temperature‐dependent costs on body size and reproductive output were relatively small (Figure 6). The biggest differences emerged in highly productive environments with dramatic increases in costs associated with higher temperatures. Specifically, when average temperature increased by 10 degrees (e.g., from 11.85 to 21.85°C or from 16.85 to 26.85°C), maximum body sizes were 3–10% smaller, with the largest differences occurring when κ=5 (specific values in Figure 6 are reported in Table S1). At the same time, these increases in temperature led to concomitant reductions in lifetime reproductive output that ranged from 3% to 10% (Figure 6b). However, it is important to note that comparing thermal increases in 5‐degree intervals only generated minor (3%–5%) decreases in growth and reproduction, unless both predators and prey were scarce (κ≤0.5, Table S1).

The interacting effects of temperature and productivity on body size showed that when both prey and predators were abundant, the benefits of growing large outweighed associated increases in metabolic costs. Remaining smaller in thermally costly conditions (to minimize energetic requirements for maintenance) equated to an increased risk of mortality through predation and thus shorter lifespans. In other words, the effects of metabolic costs on lifespan were indirectly expressed through body size.

To understand the robustness of the main results in Figures 5 and 6, we conducted sensitivity analyses. First, we varied the parameter *h*, representing predator efficiency (Equation 3; Andersen, 2019), to change the allometric properties of the risk of predation (Figure S6). However, while varying this parameter affected maximum lifespan by changing the risk of predation, it did not dramatically change individual growth trajectories or patterns of size‐specific reproduction (Figure S6). By contrast, the constraint on the amount of lipid stores that can be spent each month on reproduction, *φ*, was of more importance for growth because it depended directly on the individual's structural mass. This parameter contributes biological realism to the state dynamics because it represents a limit on the maximum possible fat stored as gonadal tissue (i.e., so fish cannot remain small and instead channel all surplus energy toward reproduction with no constraint; at some point, their body cavity will limit gonadal tissue). However, it is difficult to measure or estimate directly, especially for fish that spawn multiple times per year, because often little is known about the frequency of spawning. In sensitivity analyses, we found that increased body sizes were favored if we made this constraint more stringent (e.g., decreased *φ* from 0.2 to 0.1), representing the case that individuals were able to devote less of their body cavity to gonadal tissue. This pattern held at both high‐ and low‐productivity values (Figure S7). Finally, we used estimates of parameters for the metabolic cost function that were measured experimentally for tuna species, because of our interest in explaining diversity in tuna growth patterns (Clarke & Johnston, 1999). However, to understand if the relatively small effect of increased temperatures on body size in Figure 6 was robust to differences in metabolic costs, we conducted sensitivity analyses in which the shape and allometric scaling of costs varied (Figures S8 and S9). We confirmed that our choice of parameters for this function had relatively minor influences on growth and reproduction.

**FIGURE 7:**
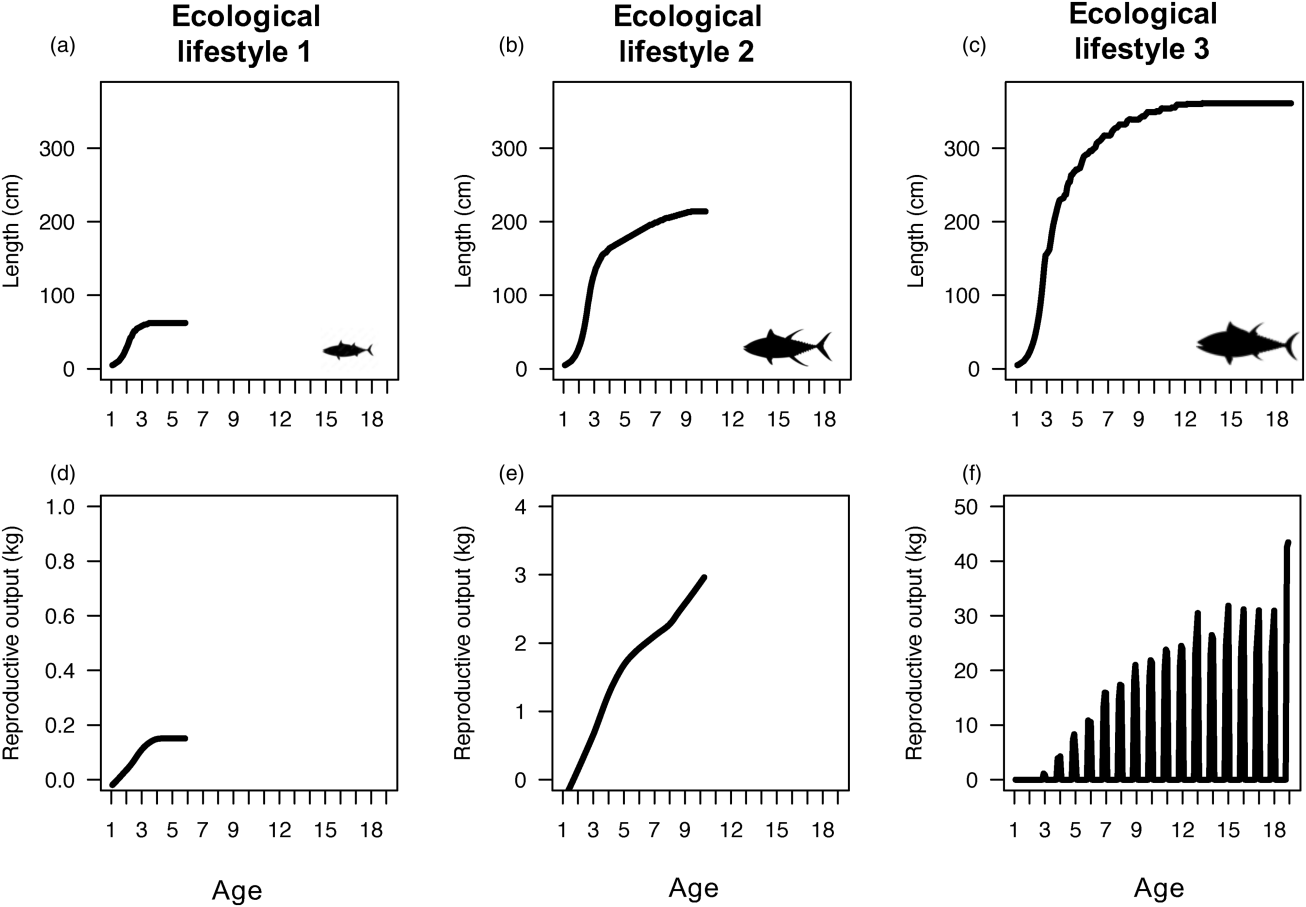
Growth (top row) and reproduction (bottom row) predicted by our general energy‐based model for the three characteristic ecological lifestyles of tunas using three representative environmental scenarios (columns). Details of each scenario and species are in Table 2. With the exception of c and f, all curves end at the age when less than 3% of the population is expected to survive, representing the cohort lifespan. a and d represent tropical shallow‐diving tuna species such as the frigate tuna *Auxis thazard*. b and e represent tropical deep‐diving tuna species such as yellowfin tuna *Thunnus albacares*. c and f represent temperate deep‐diving species such as Atlantic bluefin tuna *Thunnus thynnus*, which migrate seasonally from higher latitudes with colder and productive waters to less productive and warmer waters. At age 18, more than 10% of individuals in the temperate deep‐diving lifestyle were still alive and are likely to live much longer (c, f). As in Figure 5, the reproductive output in panels d and e was smoothed using the loess function.

In the environmental scenarios representing the three ecological lifestyles of tunas (Table 2), our model predicted growth, body size, and reproductive patterns that were qualitative matches with typical species of each of the three tuna ecological lifestyles (Figure 7). With the environmental scenario representing the tropical shallow‐diving lifestyle, we predicted a maximum body size of 70 cm, longevity of 6–7 years of age, and continuous reproduction at relatively low levels (left column, Figure 7, Table 2). These predicted traits are similar to those of frigate tuna *Auxis thazard*, a tropical shallow‐diving tuna species with small body size. With the environmental scenario parametrized to match the tropical deep‐diving lifestyle, we predicted growth to sizes well over 200 cm, lifespans of less than 15 years, and continuous reproductive output that increases over the individual's life (middle column, Figure 7, Table 2). While we believe this is broadly consistent with the life histories of tropical deep‐diving tuna species such as yellowfin tuna *Thunnus albacares*, lifetime reproductive patterns of these batch‐spawning species are not well‐known (Horswill et al., 2019). In the environmental scenario matching the lifestyle of temperate deep‐diving tunas, such as Atlantic bluefin tuna, *Thunnus thunnus*, we predicted the maximum body size exceeded 350 cm, a longevity that was longer than 18 years (right column, Figure 7, Table 2). Spawning was constrained to be seasonal in tunas in the third ecological lifestyle (i.e., spawning could only occur in temperatures above 24°C, which we specified individuals experienced for 3 months of the year).

### 3.1 Analysis of seasonality in temperature and spectrum productivity

The results of the tuna case study yielded an unexpected pattern: when spawning activity, costs associated with increased temperature, and ecosystem productivity varied seasonally (i.e., in the third ecological lifestyle of temperate deep‐diving tunas), individuals grew to be substantially larger than the size observed under constant environment conditions that are otherwise comparable in terms of average productivity (κ) and temperature (τ).

**FIGURE 8:**
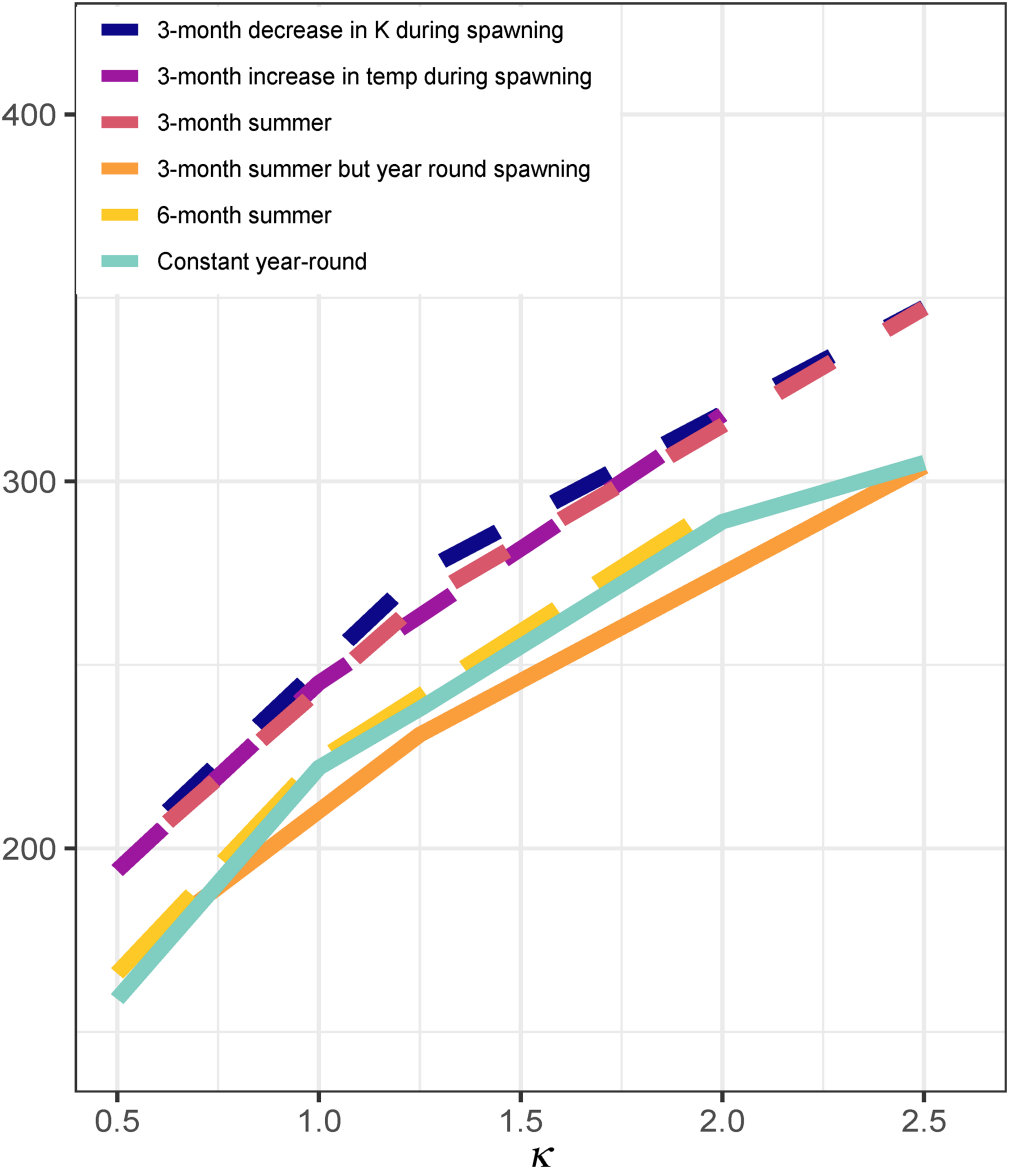
Maximum body length across values of κ in multiple scenarios with varying seasonality in food, temperature, and spawning season duration. As described in the text, solid lines represent scenarios where spawning is possible every month of the year, while the dashed lines represent scenarios where spawning is restricted to 3 months (short‐dashed) and 6 months (yellow long‐dashed) of the year. Colors correspond to different scenarios in which temperature and productivity fluctuate temporally.

To investigate this pattern further, we made several subsequent comparisons of the effects of seasonality in spawning season length, temperature (in which species spawned for 3 months in waters that are 9 degrees warmer than their winter foraging grounds) and productivity (which increased threefold during the winter months spent on the foraging grounds). In Figure 8, for a range of ecosystem productivities (κ = 0.5–2.5 when averaged across the 12‐month period), we compared the maximum lengths predicted in constant environments with scenarios in which reproductive season length varied (represented by the dash length in Figure 8), and/or temperature and productivity fluctuated over the year, for a total of six contrasting scenarios: (i) the most basic constant scenario when temperature, productivity (κ), and spawning were constant year‐round (solid aqua line in Figure 8, which is identical to the τ = 16.85°C scenarios shown in Figure 6); (ii) a seasonal scenario when temperature increased during the 3‐month spawning season, but productivity κ did not change throughout the year (purple short‐dashed line in Figure 8), (iii) a seasonal scenario with a threefold increase in productivity for 9 months to represent time spent in foraging grounds, but with temperature kept constant throughout the year (navy blue short‐dashed line in Figure 8) (iv) the full seasonal scenario where productivity and temperature varied during the 3‐month summer when spawning was possible (salmon‐pink short‐dashed line in Figure 8; note this scenario is similar to Figure 7c, but with lower values of κ); (v) a full seasonal scenario where the warm, low‐productivity season lasted for 6 months instead of 3 (yellow long‐dashed line in Figure 8); and (vi) a seasonal scenario where productivity and temperature varied during the 3‐month summer, but spawning was possible during the full 12 months (solid orange line in Figure 8).

These comparisons clarified that the restriction of spawning to 3 months of the year has the largest effect on maximum body size across a range of ecosystem productivities (because the three short‐dashed lines are substantially greater than solid and long‐dashed lines in Figure 8). When spawning was restricted to 3 months, the effects of temperature‐associated costs and productivity alone were comparable to those when both effects are combined (in Figure 8, the three short‐dashed lines are similar). When spawning was restricted to 6 months of the year, and temperature and productivity fluctuated seasonally, individuals grew to intermediate sizes. Smaller sizes were predicted when spawning was possible during every month of the year, especially when temperature and productivity fluctuated seasonally (the solid orange line in Figure 8).

From this set of follow‐up analyses, we concluded that resources and temperature effects on energy budgets were not the primary drivers of larger body sizes in our seasonal model. Rather, the compression of reproductive opportunities into 3 months of the year, and the temporal separation of reproduction from the most valuable foraging opportunities, favors increased allocation to growth in seasonal environments.

## 4 DISCUSSION

We used state‐dependent energetic modeling to test our hypothesis that accounting for trophic interactions represented by size spectra and the effects of seasonality in metabolic demands and spawning opportunities can explain observed diversity in life histories of fishes. We then asked whether variation in specific environmental conditions, including seasonality in resources and metabolic costs associated with temperature, can predict the observed life history traits of three tuna species representing different ecological lifestyles. By unpacking the effects of temporal variation in spawning opportunities, metabolic demands, and foraging opportunities, we found that seasonal cycles in resource intake and expenditures have a greater effect on the evolution of body size than that of direct metabolic costs arising from warmer temperatures. Our results do not directly address potential effects of temperature on environmental productivity and individual activity levels, both of which can increase consumption. However, we found that when individuals experienced the same metabolic costs with differing levels of ecosystem productivities, increasing productivity has a positive effect on growth rates. This contrast suggests that net effects of temperature on growth depend on both metabolism and resource availability.

The key difference between our general energetic model and other models of life history evolution of organisms in varying environmental conditions (Ejsmond et al., 2010; Kozłowski & Teriokhin, 1999; Lika & Kooijman, 2003) is that we consider the mass‐specific scaling of metabolic costs with temperature in addition to differences in primary productivity, which drive changes in predator–prey interactions. Across a range of temperatures, our model predicted the benefits of growing large, though the size spectrum outweighed the increase in metabolic costs until very large sizes and/or very dramatic increases in temperature. For example, although our model predicted smaller body sizes and decreased lifetime reproduction due to increased costs at higher temperatures when both predators and prey were scarce, these differences were relatively small – all else being equal – until average temperatures increased by 10–15°C. However, increased temperatures may also affect the productivity of ecosystems, offsetting or exaggerating these costs (Lotze et al., 2019). Consistent with expectations, our model predicted larger maximum body sizes, greater reproductive output, and longer lifespans in more productive ecosystems. The parameter representing productivity, κ, had a much stronger influence on our results than any of our other parameters, with one exception being the estimation of age‐specific mortality. Under all scenarios, the realized mortality at each age was more sensitive to parameter values determining predator efficiency in capturing prey (*h*, Equation 3) than it was to κ. However, while this parameter changed survival, it did not substantially affect growth trajectories or reproductive patterns.

Our results provide a novel explanation for asymptotic growth of fishes. Prior work has predicted asymptotic growth patterns based on physiological arguments for intrinsic geometric constraints (Pauly, 2010; von Bertalanffy, 1960) or by specifying that increased allocation to reproduction limits growth as organisms age (Jusup et al., 2011). In our model, multiple (mainly extrinsic) ecological mechanisms led to the slowdown of growth and emergence of an asymptotic size in each environment. Unlike previous research, in our results, the onset of reproduction alone did not correspond immediately to decreasing growth. Instead, the availability of prey, the physical and temporal limits on resource intake and storage, the risk of predation, and constraints on gonad size and reproductive timing all increased the advantage of growing to larger body sizes; these advantages balanced against metabolic demands, and the actual energy requirements of growth determined maximum body sizes.

We used variation among the ecological lifestyles of tunas to motivate our model parameterizations and comparisons, while also aiming to provide general insights into the evolution of fish life histories. We successfully predicted growth and reproductive patterns that are consistent with species representing each ecological lifestyle of tunas (Figure 7). Unexpectedly, we found an effect of seasonality on growth patterns. Seasonal variation in mortality has been shown to affect body size (Kozłowski & Teriokhin, 1999), but in our model, mortality depended only on size and did not differ among seasonal environments. Further investigation revealed this effect was robust to fluctuations in metabolic demands or in productivity alone and instead depended on the spawning season duration. Our findings that large body sizes emerged from the optimal allocation strategy in seasonal environments – whether or not temperature varied – offer a novel explanation for latitudinal gradients in body size (Verberk et al., 2021). The analysis in Figure 8 supports the conclusion that growth to larger maximum body sizes is driven by the limited time available for reproduction, coupled with the opportunity to forage and store energy in productive ecosystems the rest of the year. These results could explain gigantism of fishes in highly seasonal polar environments. Note that the mechanism here contrasts proposed explanations for latitudinal clines in arthropod body sizes, which also invoke season length (Blanckenhorn & Demont, 2004; Horne et al., 2015), because for these species the growing season is shorter, and thus generation time is shorter, at high latitudes.

Our model illustrates that predicting responses in growth and reproduction in changing environments is complex because optimal strategies will respond to selective pressures from many factors, including intrinsic constraints on storage and reproduction, physiology, and the nature of ecosystem size spectra. For fishes like tunas, which often exhibit a combination of capital and income breeding, and for which reproduction is constrained by ambient temperature, we expect that as climate change generates warmer conditions, eventually spawning at higher latitudes for longer periods could be possible. However, our results suggest that cascading effects on growth are not easily predicted and will depend on how warming waters reverberate through the size spectrum.

Future work clarifying the complicated relationship between the productivity of oceanic ecosystems and temperature is needed because outcomes are highly uncertain (Lotze et al., 2019). Changes in climate over recent decades have been shown to affect fish recruitment, growth, and fishery productivity (Free et al., 2019; Oke et al., 2020). Several mechanisms have been proposed to explain these changes (Fernandes et al., 2020). Early models based on differences in resource intake and metabolic demands predicted that increased temperature will lead to reduced growth and smaller body sizes, i.e., ‘shrinking fishes’ (Cheung et al., 2008), but experimental evidence shows growth is less affected by temperature than reproductive allocation (Wootton et al., 2022) and that metabolic rate can adapt to increased temperatures (Pilakouta et al., 2020). Both physiological models and experimental observations have necessarily minimized confounding effects of environmental productivity, seasonality, and ecological interactions. Our model includes some of the extrinsic abiotic and ecological variables that previous work has had to ignore, but we focused primarily on the effects of temperature on metabolic costs in this framework and determined that these costs had only minor effects on growth (Figure 6). Our results imply that the negative effects of temperature on metabolic costs are relatively unimportant compared to the overall productivity of ecosystems, as well as seasonal dynamics of resource acquisition. Yet direct effects of temperature on ecosystem productivity could drive variation in growth and reproduction (Figure 5). Temperature can change activity levels of both predators and prey, as well as physiological processes such as digestion efficiency. Including such processes in future investigations of our questions, while increasing model complexity, could be useful to address biological responses to ongoing environmental change.

The current model has several additional assumptions that could be explored in future analyses. In the analyses presented here, we have assumed a static relationship between length and mass and the conversion of mass to joules (energy density of tissue). Keeping these relationships constant ensured a common currency linking individual energy budgets to ecological changes in foraging success and predation risk. Exploring the consequences of these relationships for differences among clades would be a natural follow‐up to our study. Furthermore, while we did not directly address the interaction between fishery‐induced selection and growth (Audzijonyte et al., 2016), it would be a rich area of further investigation. Plastic or evolutionary responses to fishing potentially include faster growth, earlier maturation rates, and smaller maximum body sizes, but the consequences for marine food webs are complex (Hočevar & Kuparinen, 2021). The framework introduced here potentially could capture how eco‐evolutionary feedbacks between fishing mortality, length and mass relationships, and ecosystem size spectra interactively affect trends in body sizes of focal species. Studies of the interaction between fishing and size spectra have been focused on lake systems where food webs have been studied in greater detail (Perälä & Kuparinen, 2020). Understanding how these physiological and ecological processes are mechanistically linked is necessary to understand how species will respond to different environmental conditions in future oceans.

In summary, our findings suggest that when predicting future growth patterns under projected changes in climate, ecological and environmental factors – primary production, prey size and availability, and predation risk – will all play a greater role than metabolic demands in determining trends in maximum body size. Nevertheless, we offer our model as a step toward models of fish growth with higher fidelity to nature that incorporate not only physiology and energy allocation budgets but are also embedded in ecosystem size spectra. We hope that further exploration of our approach may lead to reconciliation of divergent results regarding the effect of climate change on fish body sizes.

## CONFLICT OF INTEREST STATEMENT

None.

## Supporting information


Data S1.

## Data Availability

The data and code that support the findings of this study are openly available in an archived GitHub repository (Kindsvater, 2023) available at https://doi.org/10.5281/zenodo.8102868.

## APPENDIX A

### Optimization details

Dynamic programming equations (Equation 12) are constrained optimization algorithms with the purpose of determining the optimal set of behaviors or decisions that maximize a quantitative metric over time, such as lifetime expected reproductive output (fitness). A key property is that the decisions at one time affect the state variables at the next time. When solving Equation 12, we consider how the allocation decisions an individual makes during 1 month of its life affect its future size, energy reserves (lipid stores), and chances of mortality and find the set of allocation to growth and reproduction that maximizes its fitness in a given environment. We are able to calculate lifetime fitness by solving the equation using backward iteration. By starting at t=T–1, and assuming there is no possibility of future fitness (i.e., the second term of Equation 12 is equal to 0 for all possible values of length and lipid stores), we can populate the array with the fitness of all possible combinations of length, lipid stores, and allocation strategies at t=T–1; this fitness will be the first term on the right side of Equation 12. This process is repeated, working backward, until *t =* 1. We can thereby determine the combination of *g* and *r* that maximizes both current fitness at *t*, and the fitness expected from t+1 until *T*, given the emergent chances of survival (which varies due to both the risk of predation and starvation), length, and lipid stores resulting from that particular strategy. Solving Equation 12 in this way produces an array storing the proportional allocation to growth and reproduction that leads to the highest lifetime fitness, for all possible combinations of size and lipid stores (l and s) for every month until the final time *T*.

Solving a dynamic programming equation with two state variables, such as Equation 12, is computationally expensive, and we employed a number of techniques to make the iteration more efficient and to approximate a smooth fitness surface. First, we constrained the parameter space that was evaluated. Specifically, we used an integer index *I* (with a maximum of $I_{\max}$) to represent lipid stores *s*, converting the index to values in joules in the dynamic loop. We then related the range of *s* we explored to each value of *l* (because lipid stores are constrained by *l*), by setting

$$s=\frac{0.6\cdot I^{3}\rho {\mathrm{al}}_{\max}^{3}}{I_{\max}^{3}}$$

In other words, we adjusted the numerical step size for possible values of *s* to be finer for smaller individuals. Furthermore, we used linear interpolation of state values (Clark & Mangel, 2000) when computing expected future fitness in Equation 12 to minimize discontinuities on the fitness landscape arising from the integer index of the energy state. We did not interpolate length since its unit (centimeters) was sufficiently fine‐grained that there were minimal effects of discontinuities.

We found that sharp transitions in the fitness landscape, such as at threshold values representing constraints, hindered our optimization algorithm. Therefore, we used an asymptotic function for the fitness increment associated with a given level of reproductive output. We let $R\left(r,s,l,t\right)$ denote the potential increment in fitness in month *t* when $L\left(t\right)=l,S\left(t\right)=s$, and a fraction of stores *r* is allocated to current reproduction and assumed that R increased smoothly toward the maximum possible for the given length *l*:

Rr,s,l=rs1+rsφwlf

The asymptotic value of this function depends on the value of *f* in the denominator, which controls the abruptness of the constraint on current fitness (Figure S2). In other words, the steepness of the multivariate landscape around the fitness optimum is modulated by *f*. For simplicity, we assume f=1 for all results presented hereafter. In that case, when $\mathrm{\varphi w}\left(l\right)$ ≫ $\frac{\mathrm{rs}}{\rho },R\left(r,s,l\right)\sim \mathrm{rs}$ and when rs ≫ $\mathrm{\varphi w}\left(l\right),$ then $R\left(r,s,l\right)\sim \mathrm{\varphi w}\left(l\right)$.

All programming was done using R 4.1.1. We ran the code to solve the dynamic programming equation and simulate the individual life histories using Rscript commands from the Linux shell of a 2019 MacPro with 16 cores and 96 GB of RAM. Jobs were run in parallel, and the runtime of each job was between 60 and 120 min. The results of each job are presented in the figures summarizing growth, reproduction, and survival data; these analyses and figures were produced in the Rstudio IDE.
